# Effects of nano-cerium dioxide on intestinal microflora in rats by oral subchronic exposure

**DOI:** 10.1371/journal.pone.0298917

**Published:** 2024-02-29

**Authors:** Qianru Ye, Dantong Jia, Jun Ji, Yang Liu, Gang Wu

**Affiliations:** 1 Department of Basic Medicine and Forensic Medicine, Baotou Medical School, Inner Mongolia University of Science and Technology, Baotou, China; 2 Clinical Laboratory, the Second Affiliated Hospital of Baotou Medical College, Baotou, China; 3 The Southern University of Science and Technology, Shenzhen, China; National Research Centre, EGYPT

## Abstract

**Objective:**

To investigate intestinal toxicity in rats and the effects of Nano-cerium dioxide on intestinal flora in rats after oral sub-chronic exposure.

**Method:**

Forty healthy male SD rats were randomly divided into four groups: a control group (deionized water) and three groups treated with different doses of Nano-ceria (e.g., 20 mg/kg, 100 mg/kg, and 500 mg/kg), with 10 rats in each group. The rats were given intragastric administrations (every other day) for 90 days. After the last intragastric administration, fresh fecal samples were collected by pressing the abdomen, and the animals were sacrificed. Jejunum, ileum and cecum tissues were retained for pathological analysis by Hematoxylin-eosin staining. The stool samples of rats were sequenced by the Illumina NovaSeq sequencing platform, and the sequencing results were further analyzed by QIIME2 software.

**Results:**

The histopathology results show that compared with the control group, in the middle- and high-dose groups, epithelial tissue was shed, lamina propria glandular structures were damaged or disappeared, and large numbers of inflammatory cells were distributed in the mucosa. The intestinal flora results show that there were no significant differences in the α-/β-diversities in each Nano-ceria-treated group compared with the control group (*P*>0.05). Compared with the control group, the intestinal pathogenic bacteria, Mucispirillum and Streptococcus increased significantly after Nano-cerium dioxide ingestion, while Weissella decreased. The abundances of Akkermansia in all Nano-ceria-treated groups were higher than those in the control group, but the abundances decreased with increasing dose. MetagenomesSeq analysis show that, compared with the control group, the abundances of S24-7, Lactobacillus and Clostridiales in all experimental groups significantly decreased.

**Conclusions:**

The sub-chronic toxicity of Nano-cerium dioxide to rats can affect the structure and abundance of intestinal microflora, long-term exposure to high doses (>100 mg/kg) causes enteritis, but there was no significant difference in the diversity of gut microbiota. Therefore, we infer that the enteritis in rats may be associated with the relative ratios of the pathogenic bacteria and intestinal probiotics, and increased of the intestinal pathogenic bacteria can disrupted intestinal homeostasis.

## 1 Introduction

Cerium oxide nanoparticles (CeO_2_-NPs) are considered to be rare earth nanomaterials with unique redox capacities and are widely used as polishing powders, catalysts, UV absorbers and other substances. In recent years, their application potential in the field of nanomedicine has also received increasing attention. Ce^3+^ can improve free radical-induced oxidative damage by binding reactive oxygen radicals and thus shows great clinical value in treating reactive oxygen-related diseases such as neurodegenerative diseases (ischemic stroke, Alzheimer’s disease) [1,2], cancer [3], chronic inflammation [4], gastric ulcers caused by ethanol or pressure [5,6], rheumatoid arthritis [7], and chronic kidney disease [8]. In addition, the properties of CeO_2_-NP mimics can be used in enzyme-linked immunosorbent reagents to detect a variety of tumor cells [9,10]. Using the cytotoxic effects of CeO_2_-NPs in micro-ecologies with different pH levels, a multifunctional nanodrug carrier system with multiple stimulus responses and drug targeted delivery has been designed [2]. CeO_2_-NPs have k-edge values similar to those of traditional contrast materials (e.g., iodine and barium), which can be specifically accumulated in inflammatory areas and protect cells from radiation damage. To a large extent, CeO_2_-NPs improve the unsatisfactory nonspecific imaging effects of traditional contrast materials, so they have the potential to become an alternative to contrast agents [11].

The future use of nanoceria in the medical field is expected to increase gradually. After entering the body, it will have more contact with the gastrointestinal tract. In-depth study of intestinal damage after oral ingestion is a prerequisite for future biological applications. However, compared with other exposure routes (e.g., respiratory, dermal, etc.), there are fewer vivo studies of oral toxicity of CeO_2_-NPs. The main toxic effects on target organs and mechanisms of action have not been described [12–14]. Studies have reveal that only a small portion (approximately 0.06% [15]) of nanomaterials is absorbed by the small intestine into the blood circulation after oral ingestion, and most of the remaining nanoparticles accumulate in the intestine and interact with it, which in turn produce effects on intestinal tissue and intestinal flora [16]. Many nanomaterials, such as zinc oxide nanoparticles [17], titanium dioxide nanoparticles [18], and halloysite nanotubes [19], deposit in intestinal tissues after oral ingestion, resulting in intestinal epithelial cell shedding and increases in the inflammatory factors to cause enteritis. And nanomaterials entering the gut can affect the species or abundance of intestinal flora by altering the structure of intestinal flora community composition, which in turn affects host metabolism and inflammation [20–22]. Therefore, we speculate that oral ingestion of CeO_2_-NP may produce similar damages, and that it is important to determine the effect of oral ingestion of CeO_2_-NP on intestinal tissue and intestinal flora.

Using male SD rats as model animals, this study explores the effects of subchronic exposure to CeO_2_-NP on rat intestines and intestinal flora. From the perspective of intestinal microorganisms, the results of the study clarify the association between characteristic changes in the microbiota and intake of CeO_2_-NPs and pathological changes in the body and help resolve whether changes in the intestinal microbiota can be detected. Since, the effects of CeO_2_-NPs on the body can be monitored, the study can provide a basis for evaluating the biosafety of CeO_2_-NPs.

## 2 Materials and methods

### 2.1 Test materials and their characterization

The tested CeO_2_-NPs (<50 nm, purity 99.95%, lot: 700290) were manufactured by Sigma‒Aldrich Corporation (St Louis, MO, USA). The sizes and shapes of the cerium dioxide nanoparticles were examined by high-resolution transmission electron microscopy (TEM). A suspension of cerium dioxide nanoparticles was prepared with deionized water and a small amount of CeO_2_-NPs particles. Approximately 5 μl of ultrasonically dispersed suspension was dropped on a Cu net for detection.

### 2.2 Animals and treatment

Forty healthy SD male rats (166.31±9.32 g, whose animal quality certificate is No. Beijing SYXK 2017–0033), were purchased from Beijing Vital River Experimental Animal Technology Co. Ltd, and housed in polythene cages under specific pathogen-free conditions. During the experiment, the rats were allowed to eat and drink freely, and the room temperature was maintained at 20–25°C with a relative humidity of 40%-60%. The dark room treatment was conducted for 24 hours. Rat cages and water bottles were numbered accordingly to prevent cross-contamination among the treatment groups.

After a week of adaptive feeding, the rats were randomly divided into four groups: a control group (deionized water) and three groups of different doses of nano ceria (e.g., 20 mg/kg, 100 mg/kg, and 500 mg/kg), with 10 rats in each of the latter groups (3 or 4 rats per cages). All rats were housed in the same room during the experiment. The CeO_2_-NP samples were diluted in deionized water and used for the animal exposures. The rats were given intragastric administrations (every other day) for 90 days, weighed once every 15 days, and fresh fecal samples were obtained by pressing the abdomen after the last gavage. At the endpoint (24h after the last gavage), mice were sacrificed under the 3% pentobarbital sodium, and all efforts were made to minimize suffering. Tissue samples (e.g., jejunum, ileum, and cecum) were collected to conduct pathological analyses. Stool samples of rats were sequenced by the Illumina NovaSeq sequencing platform, and the obtained sequences were further analyzed by QIIME2 software.

All procedures used in this experiment were reviewed and approved by the Medical Ethics Committee of Baotou Medical College (No. 2018016). The animal experiments were carried out at the Animal Center of Baotou Medical College in accordance with the Guidelines of the Ethical Committee of Experimental Animal Center of Baotou Medical College and the National Guidelines for Experimental Animal Welfare.

### 2.3 Index detection

#### 2.3.1 Histological evaluation

**Fig 1:**
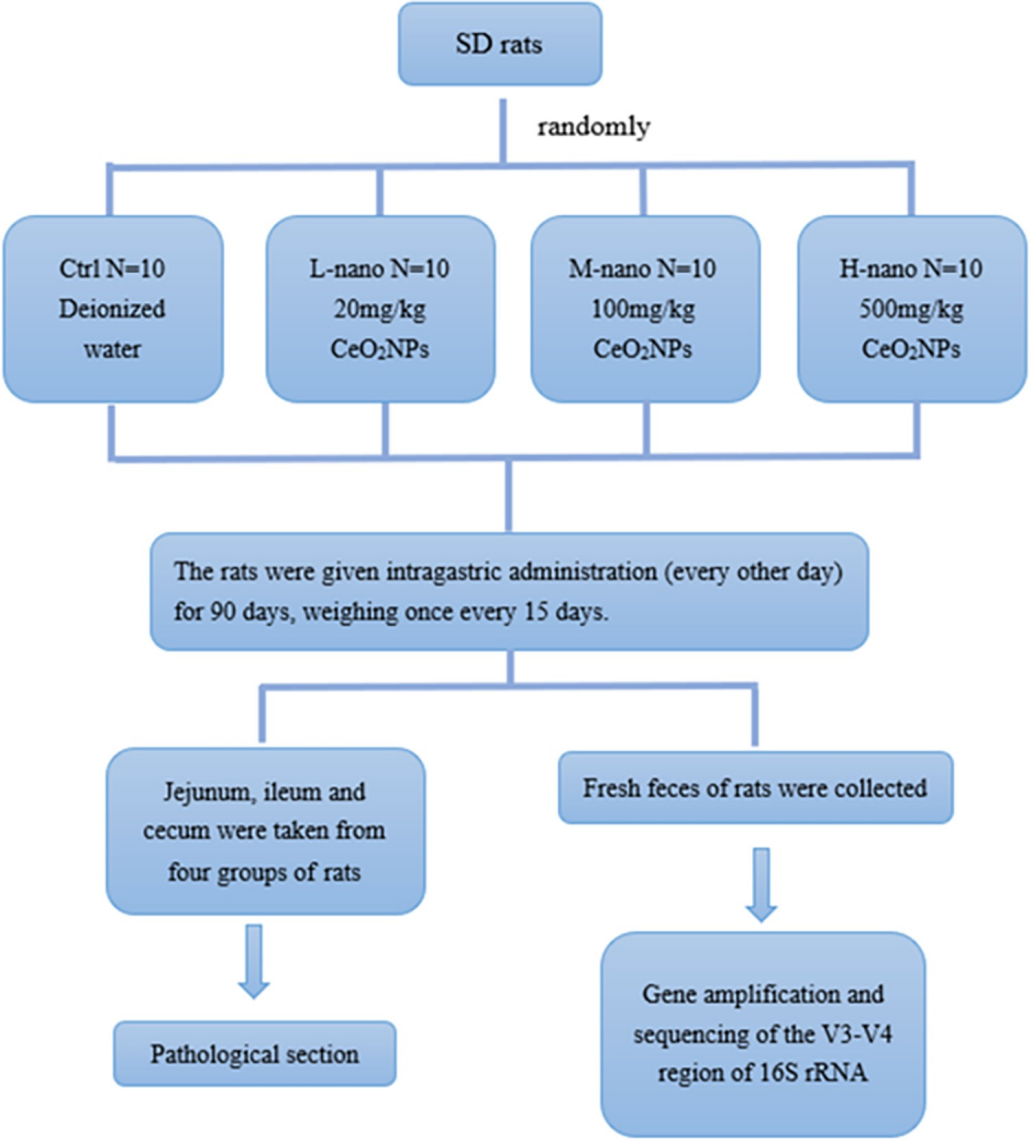
Flow chart of the experiment.

The rat jejunum (approximately 10 cm), ileum (approximately 3 cm from the cecum junction) and tip of the cecum were longitudinally cut, and the contents were washed with sterile saline. Approximately 6 mm was cut and trimmed until the upper and lower sections were smooth, and then fixed in 4% paraformaldehyde tissue fixing solution (the tissue should be kept in a tubular structure) for more than 48 hours. Gradient dehydration, paraffin embedding, sectioning (thickness: 5 μm), and HE staining were performed to observe pathological changes in these tissues under a microscope. Two histopathologists blindly evaluated the samples and provided their results. The experimental flow chart is shown in Fig 1.

#### 2.3.2 16S rRNA gene sequencing

Shanghai Personal bio Co. Ltd. helped to complete the extraction and detection of DNA from fecal samples, PCR amplification in the 16S V3-V4 zone, and high-throughput sequencing. Samples were extracted from DNA using the Omega M5635-02 kit, and the extractions were conducted according to the kit instructions. DNA was also quantified by spectrophotometry, and the DNA extraction quality was detected by 1.2% agarose gel electrophoresis. The primers used for PCR amplification were as follows: 338F (5’-ACTCCTACGGGAGGCAGCA-3’) and 806R (5’-GGACTACHVGGGTWTCTAAT-3’). The amplification reaction system was as follows: 5×buffer 5 μL; dNTP (2.5 mM) 2 μL; forward primer (10 μM) 1 μL; reverse primer (10 μM) 1 μL; DNA template 1 μL; ddH2O 14.75 μL; and Fast pfu DNA Polymerase 0.25 μL. The parameters for the amplification reaction are shown in Table 1.

**Table 1:** PCR reaction parameters.

	Temperature	Time
	98°C	5min
24 cycles	98°C	30sec
	52°C	30sec
	72°C	45sec
	72°C	5min
	12°C	……

The number of amplification cycles should be strictly controlled, and a negative control should be arranged. The obtained amplification products were used to prepare sequencing libraries using Illumina’s TruSeq Nano DNA LT Library Prep kit. After a strict quality check, 2×300 bp pair-end sequencing was performed using a NovaSeq-PE250 sequencer, and the sequencing lengths of the target fragments were 200–450 bp to meet the sequencing quality.

#### 2.3.3 Bioinformatics analysis of microbiota

In this study, high-throughput sequencing was performed on 40 rat fecal samples that were obtained after 90 d of CeO_2_-NP ingestion, which yielded a total of 5127574 downstream raw sequences (with primers removed). The sequences were quality-controlled, denoised, spliced, and dereplicated using the DADA2 [23] method (equivalent to clustering at 100% similarity), after which characteristic sequences (ASVs) were obtained. For each ASV, Selected Greengenes database, the classify-sklearn algorithm in the QIIME2 platform was used, and species annotation was performed using a pretrained naive Bayes classifier. Based on the clustering results, α-diversity analysis and β-diversity analysis were performed and based on the annotation results, information on species compositions at each taxonomic level was obtained.

### 2.4 Statistical analysis

Statistical analyses were maded using SPSS 23.0. The one-way analysis of variance (ANOVA) was used to compare the rat weights. The Wilcoxon rank-sum test was used in the bioinformatics analysis,such as alpha diversity analysis and species difference analysis. Beta diversity was used as a measure for the microbiota structure between groups in unweighted UniFrac analysis, and analyses of similarities (PERMANOVA) were performed using the QIIME2. The α-diversity and β-diversity indices were calculated based on the rarefied ASVs counts using the QIIME2. A result was considered statistically significant at *P* <0.05.

## 3 Results

### 3.1 Characterization results of CeO_2_-NPs

**Fig 2:**
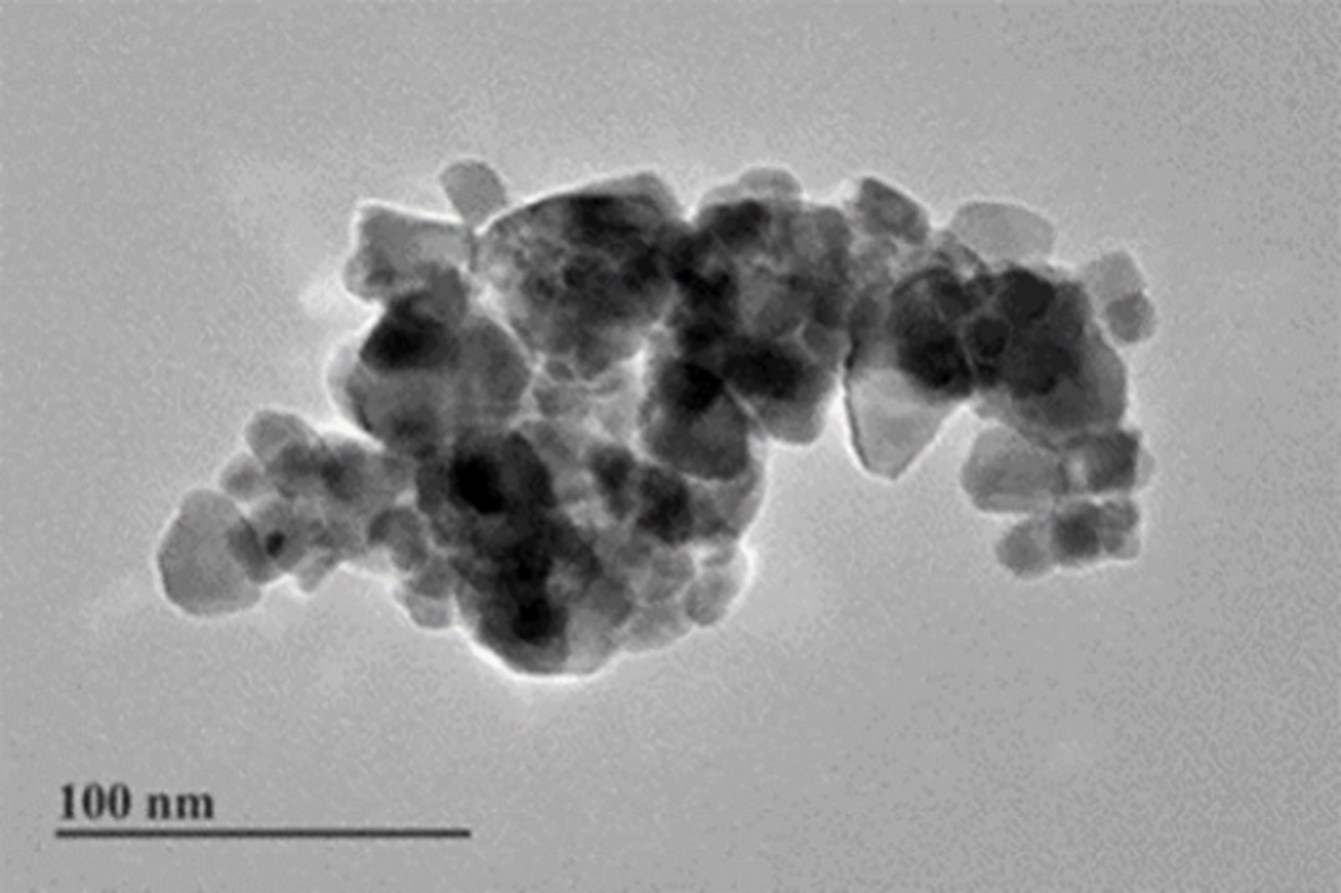
CeO_2_-NPs TEM characterization results.

According to the transmission pattern observed with the electron microscope, a single CeO_2_-NP particle has a lens diameter of < 50 nm, the shape is similar to a cube, and the particles exhibit agglomeration (Fig 2).

### 3.2 Toxic effect of CeO_2_-NP ingestion on rats

#### 3.2.1 Weight and general condition

**Fig 3:**
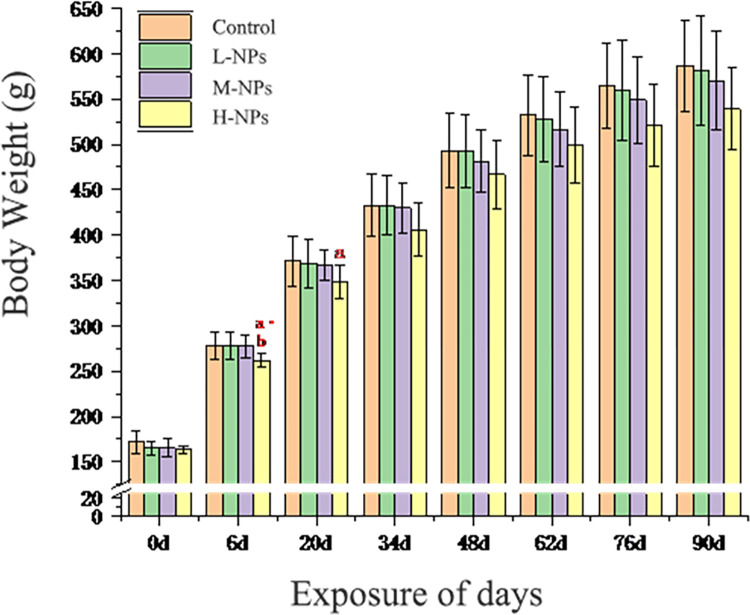
Changes in body weight of rats after subchronic exposure to CeO_2_-NPs. Data represent mean weight ± SD. L-NPs = 20mg/kg bw/day CeO_2_-NPs, M-NPs = 100mg/kg bw/day CeO_2_-NPs, H-NPs = 500mg/kg bw/day CeO_2_-NPs. Compared with the vehicle control, a means a significant difference at the *p* < 0.05 level, a· means a significant difference at the *P*<0.01, Compared with the low-dose group, b means a significant difference at the *P*<0.01.

During ingestion, the rats were in good condition, and no adverse reactions or deaths were observed. As shown in Fig 3, compared with the control group, there was no significant differences in rat weights in the low-dose group, while the rat weights in the medium-dose and high-dose groups showed a downward trend. However, the differences were not statistically significant except for the 6th and 20th days of gastric lavage in the high-dose group.

#### 3.2.2 Intestinal histopathological findings

**Fig 4:**
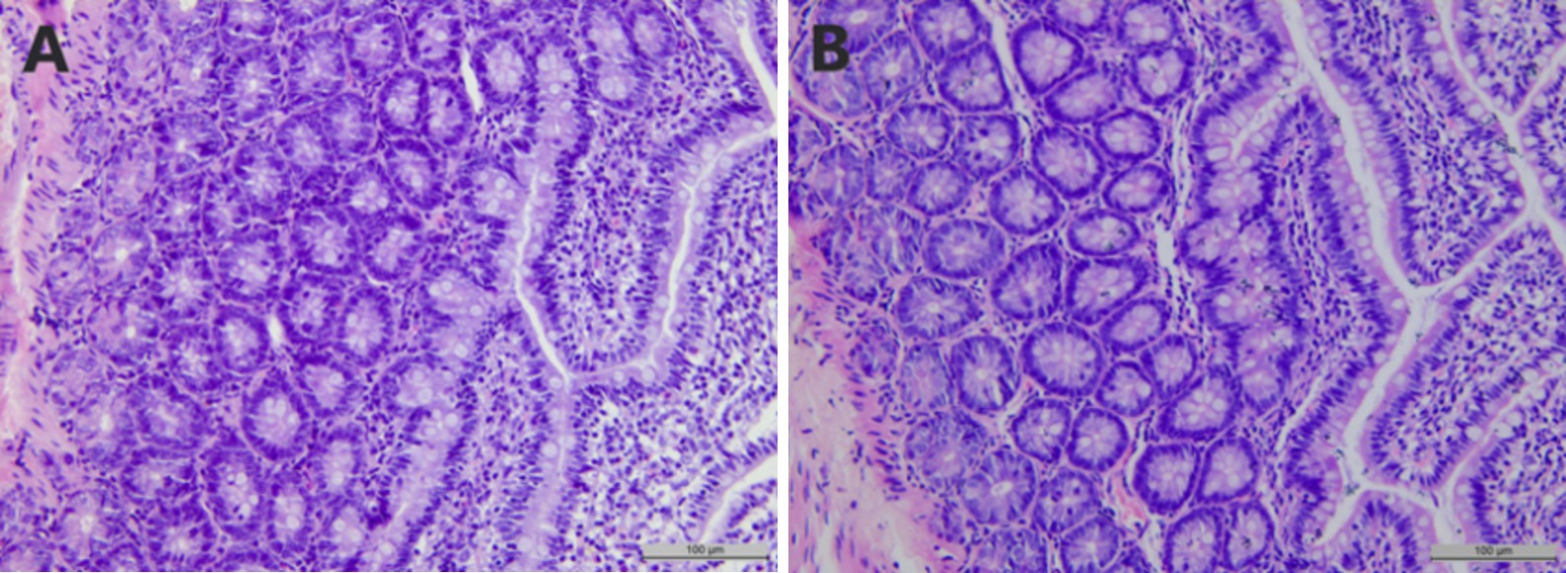
Effects of subchronic exposure to CeO_2_-NPs on jejunum tissue in rats. (A)Control group, (B) CeO_2_-NPs groups with different doses (20 mg/kg, 100 mg/kg, and 500 mg/kg).

*3*.*2*.*2*.*1*. *Pathological results of Jejunum*. As shown in Fig 4, the structures of the intestinal villi and native glands of rats were intact compared with those of the control group after CeO_2_-NP ingestion. There were no obvious differences, and no obvious pathological damage was seen.

**Fig 5:**
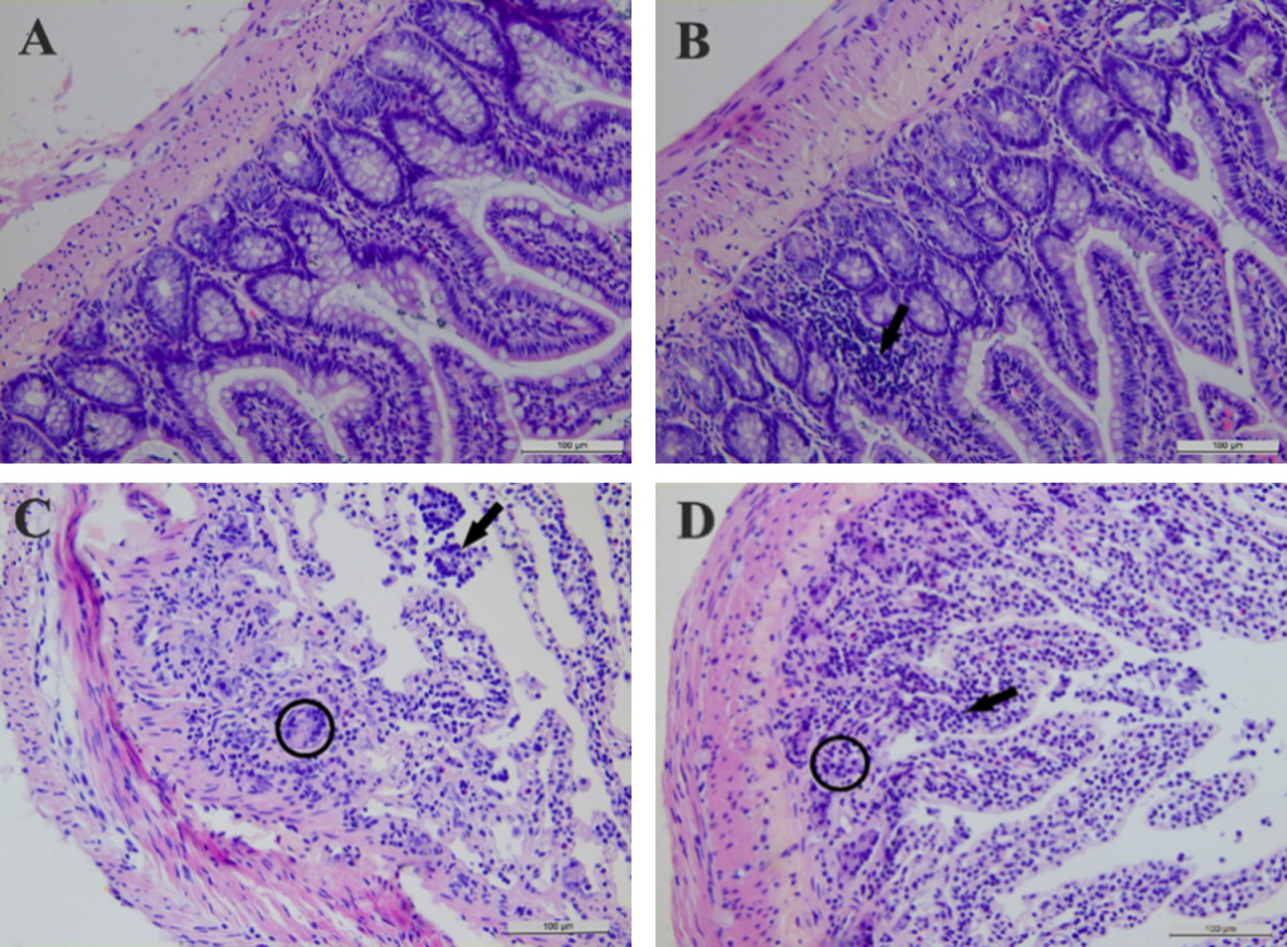
Effects of subchronic exposure to CeO_2_-NPs on ileum tissue in rats. (A) Control group, (B) 20mg/kg bw/day CeO_2_-NPs, (C) 100mg/kg bw/day CeO_2_-NPs, (D) 500mg/kg bw/day CeO_2_-NPs. Arrows indicate inflammation cells, and circles indicate broken structures of the lamina propria.

*3*.*2*.*2*.*2*. *Pathological results of ileum*. As shown in Fig 5, the structures of the intestinal villi and intrinsic glands of rats in the control group were intact, and the structures of the low-dose group were similar to those of the control group, but the mucosal layer occasionally showed small focal aggregations of lymphocytes, suggesting a mild inflammatory response. In the medium- and high-dose groups, epithelial tissue was detached, the intrinsic layer glandular structures were broken or had disappeared, and large numbers of inflammatory cells were distributed in the mucosal layer, indicating a development of chronic mucositis.

**Fig 6:**
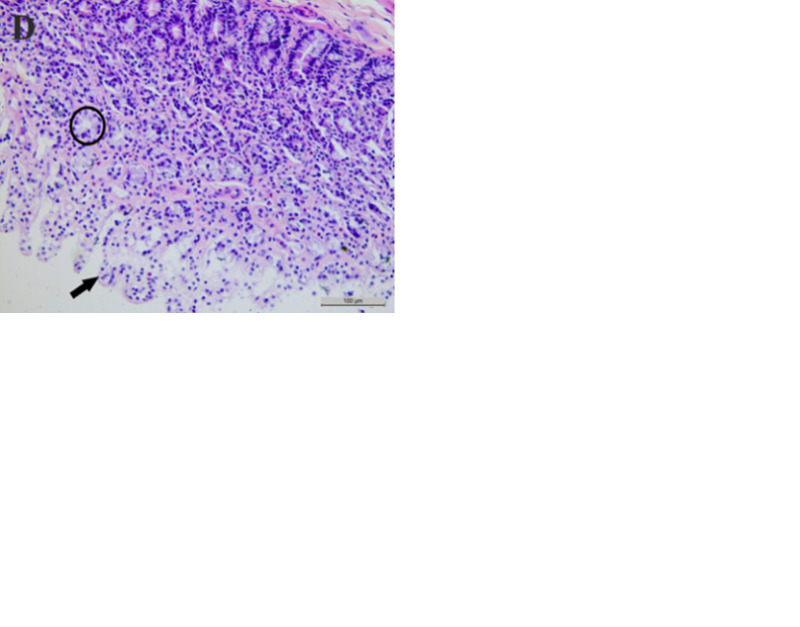
Effects of subchronic exposure to CeO_2_-NPs on caecum tissue in rats. (A) Control group, (B) 20mg/kg bw/day CeO_2_-NPs, (C) 100mg/kg bw/day CeO_2_-NPs, (D) 500mg/kg bw/day CeO_2_-NPs. Arrows indicate the mucosal layer detachment, and circles indicate broken structures of the lamina propria.

*3*.*2*.*2*.*3*. *Pathological results of Caecum*. As shown in Fig 6, the structures of the intestinal villi and intrinsic glands of rats in the control group were intact, but the structures of the mucosal layer occasionally showed detachment in the low-dose group, suggesting a mild inflammatory response. In the medium- and high-dose groups, epithelial tissue was detached, the intrinsic layer glandular structures were broken or had disappeared, and large numbers of inflammatory cells were distributed in the mucosal layer, indicating a development of chronic mucositis.

### 3.3 Effects of CeO_2_-NPs on the intestinal flora of rats

#### 3.3.1 Changes in flora structure

**Fig 7:**
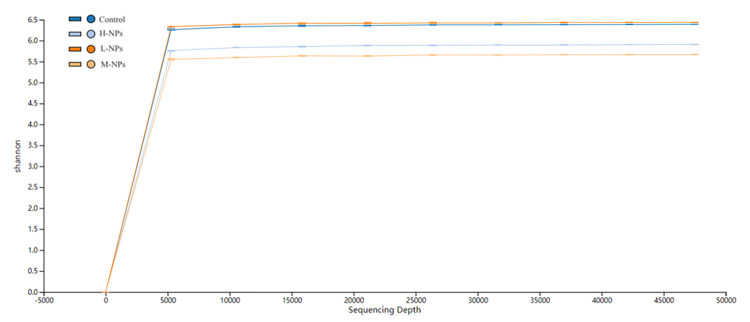
Sparse curve results. L-NPs = 20mg/kg bw/day CeO_2_-NPs, M-NPs = 100mg/kg bw/day CeO_2_-NPs, H-NPs = 500mg/kg bw/day CeO_2_-NPs.

*3*.*3*.*1*.*1 Quality control of sequencing results*. In this study, a sparse curve was plotted with the sequencing depth as the horizontal coordinate, and a number of ASVs were detected at the current depth as the vertical coordinate. As shown in Fig 7, the curve gradually flattened out when the sequencing depth reached 15000–20000, indicating that the sequencing results were sufficient to reflect the diversity present in the samples.

*3*.*3*.*1*.*2 Alpha diversity analysis*. The alpha diversity index (α-diversity) provides a description of the diversity of a community in the form of a statistical analysis. In this experiment, the diversity index was calculated by QIIME2 software, and the results are shown in Table 2. The Good’s coverage index shows that the species coverage in all the four groups of samples was above 99.5%, which again indicates that the sequencing results of this study could reflect the information of most species present in the samples. Compared with the control group, the Chao1 index and observed index were slightly lower in the low-dose group, but the differences were not statistically significant (*P*>0.05). The Pielou_e index, Simpson index and Shannon index of the experimental group were smaller by varying amounts compared with those of the control group after CeO_2_-NP ingestion, but the differences were not statistically significant (*P*>0.05). These results indicate that oral exposure to CeO_2_-NPs did not affect the α-diversity of rat intestinal flora.

**Table 2:** Effects of subchronic exposure to CeO_2_-NPs on α-diversity of intestinal microflora in rats(n = 10, $\bar{X}\pm S$).

Group	Chao1	Observed	Pielou_e	Simpson	Shannon	Good’s coverage
Control	1319.41±281.08	1204.1±279.78	0.63±0.11	0.90±0.09	6.47±1.30	99.58%
L-NPs	1177.16±355.59	1044.04±314.39	0.60±0.11	0.89±0.11	6.03±1.31	99.60%
M-NPs	1378.26±169.54	1214.97±142.55	0.56±0.07	0.85±0.09	5.75±0.71	99.50%
H-NPs	1334.45±290.18	1216.88±290.78	0.59±0.13	0.85±0.13	6.04±1.48	99.58%
*KW*	1.797	1.816	2.192	2.592	2.164	4.44
*P*	0.616	0.611	0.533	0.459	0.539	0.218

^a^The Chao1 index and the Observed index are used to describe species richness; Pielou_e index describes the uniformity of species; The Shannon index and the Simpson index describe species diversity (richness and uniformity); The Good’s coverage index characterizes the coverage of species in a community. L-NPs = 20mg/kg bw/day CeO_2_-NPs, M-NPs = 100mg/kg bw/day CeO_2_-NPs, H-NPs = 500mg/kg bw/day CeO_2_-NPs.

**Fig 8:**
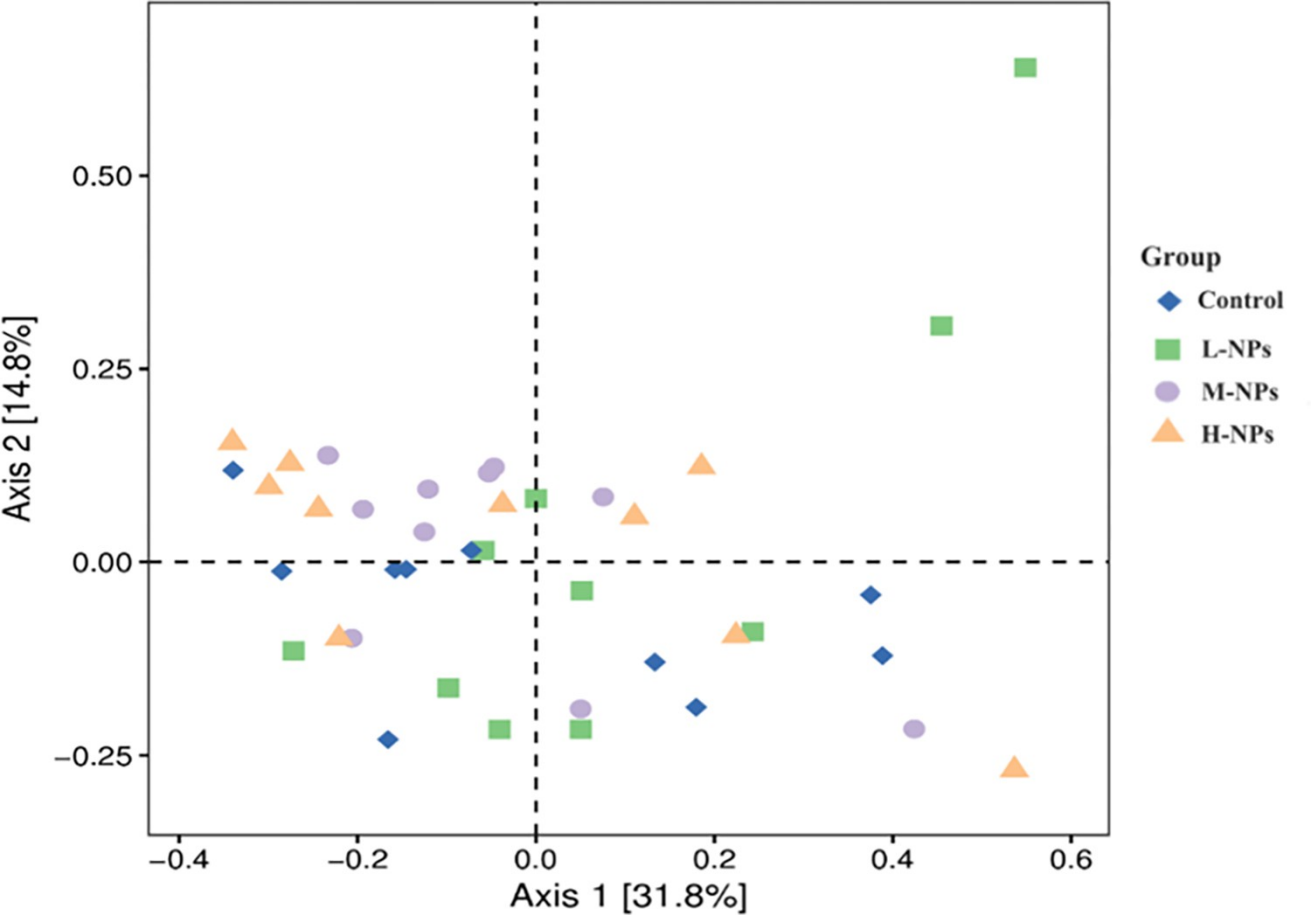
Two-dimensional ranking diagram of samples analyzed by PCoA. Each point in the figure represents a sample, and different colors indicate different groups of processes. The percentages in the axis parentheses represent how much the corresponding axis can interpret the differences between samples. Projection analysis is recommended. L-NPs = 20mg/kg bw/day CeO_2_-NPs, M-NPs = 100mg/kg bw/day CeO_2_-NPs, H-NPs = 500mg/kg bw/day CeO_2_-NPs.

*3*.*3*.*1*.*3 Beta-diversity analysis*. Beta-diversity (β-diversity) analysis was used to compare the magnitudes of differences that were present among samples in terms of species diversity. In this experiment, the fecal flora diversity data of rats in all treatment groups were analyzed by weighted UniFrac based on the principal coordinate analysis, and the 16S rRNA sequences of each sample were extracted as principal component factors and plotted on Principal coordinates analysis (PCoA) plots. The results are shown in Fig 8, and no significant separation trends were observed among the control group and CeO_2_-NP dosage groups.

*3*.*3*.*1*.*4 PERMANOVA analysis*. The PERMANOVA analysis statistically verifies the distribution patterns presented in the PCoA ranking chart. As shown in Table 3, and compared with the control group, there was no significant differences after CeO_2_-NP ingestion, indicating that the exposure of CeO_2_-NPs had no significant effects on the intestinal flora community structures.

**Table 3:** Pairwise permanova results.

Group 1	Group2	Sample	Permutations	*pseudo-F*	*p-value*	*q-value*
Control	L-NPs	20	999	0.994661	0.402	0.4884
	M-NPs	20	999	1.013399	0.365	0.4884
	H-NPs	20	999	0.852076	0.407	0.4884

#### 3.3.2 Characteristic changes in intestinal flora

**Fig 9:**
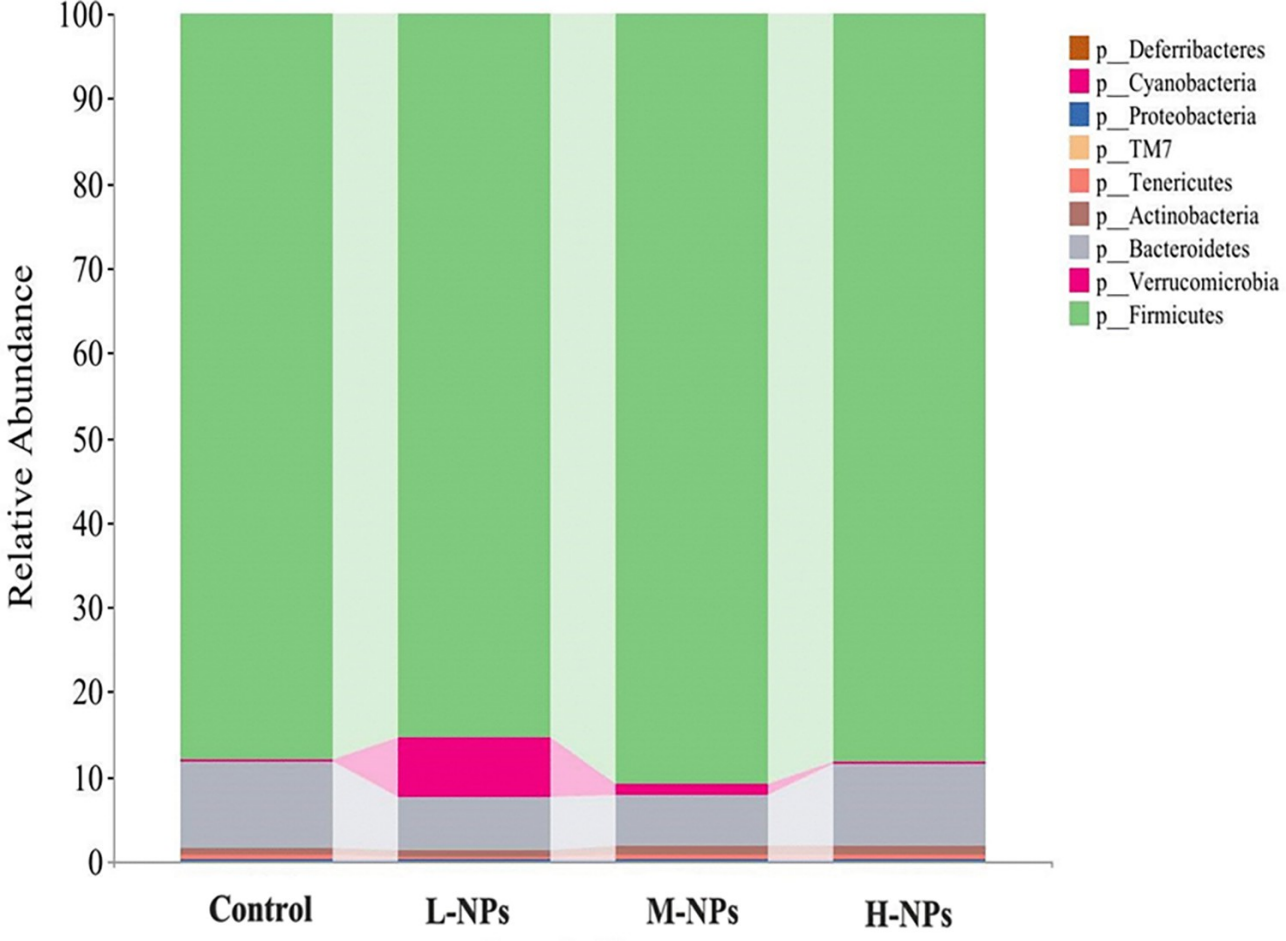
Relative gut microbiota (top nine) abundance at the phylum. L-NPs = 20mg/kg bw/day CeO_2_-NPs, M-NPs = 100mg/kg bw/day CeO_2_-NPs, H-NPs = 500mg/kg bw/day CeO_2_-NPs.

*3*.*3*.*2*.*1 The phylum level*. At the phylum level, a total of 22 different phyla were detected in the 40 stool samples obtained from the four groups, and a total of four phyla had high abundances (relative abundance >1%) (Fig 9), namely, Firmicutes, Bacteroidetes, Actinobacteria and Verrucomicrobia. Among them, the thick-walled phylum and bacteriophage phylum were the dominant phyla shared by the four groups, with the sum of relative abundances greater than 90%, occupying the dominant position in the composition spectrum of the intestinal flora. Compared with the control group, the Verrucomicrobia abundances decreased with increasing ingestion dose, and the differences were statistically significant in the low- and high-dose groups (*P*<0.05). Deferribacteres were the species that appeared specifically after CeO_2_-NP ingestion, and their abundances increased with increasing dose, with statistically significant differences in the low- and medium-dose groups compared to the control group (*P*<0.05). Further analysis of the species annotation results shows that the changes in Deferribacteres abundance in this experiment were mainly due to changes in abundance of the genus Mucispirillum; the change in abundance of Verrucomicrobia was mainly due to the change in abundance of the genus Akkermansia.

**Fig 10:**
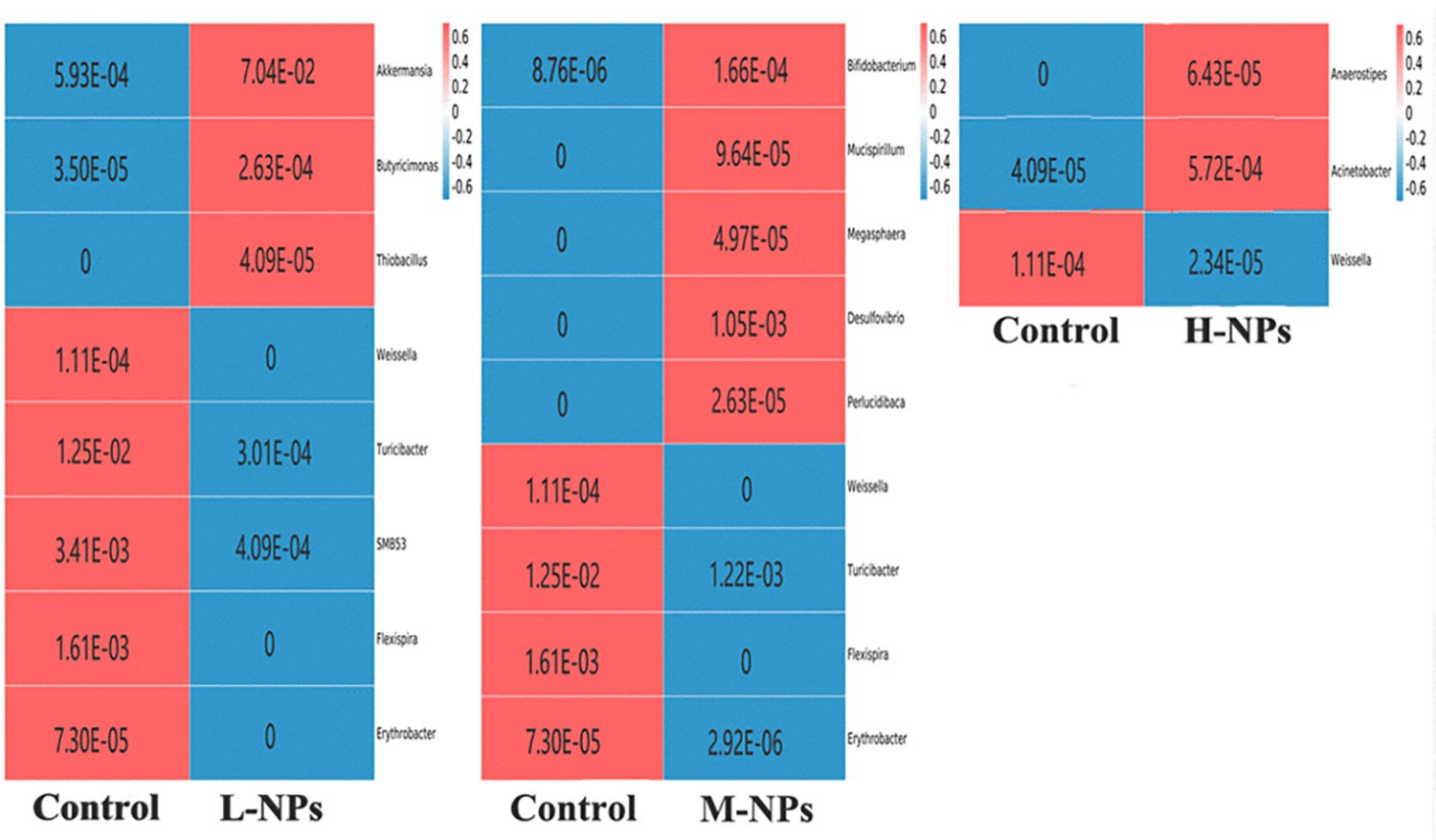
Correlation heat map of experimental group and control group(genus). L-NPs = 20mg/kg bw/day CeO_2_-NPs, M-NPs = 100mg/kg bw/day CeO_2_-NPs, H-NPs = 500mg/kg bw/day CeO_2_-NPs.

*3*.*3*.*2*.*2*. *Genus level*. The species with significant abundance differences in the flora in the experimental group compared with the control group are shown in Fig 10. at the genus level, and the results indicate that the abundance trends for intestinal flora among the groups were not identical after ingestion of CeO_2_-NPs at different doses compared with the control group. Specifically, the low-dose group showed significant increases in abundances of the beneficial intestinal bacteria, Butyricimonas and Akkermansia (*q*<0.05), and significant decreases in abundances of the potentially pathogenic intestinal bacteria, Turicibacter, SMB53 and Flexispira (*q*<0.01); the medium- and high-dose groups mainly showed significant increases in the abundances of the potentially pathogenic intestinal bacteria, Mucispirillum, Megasphaera, Acinetobacter and Desulfovibrio (*q*<0.01). In addition, compared with the control group, the relative abundance of Weissella showed a significant decrease in abundance after ingestion of CeO_2_-NPs (*q*<0.05).

*3*.*3*.*2*.*3 MetagenomeSeq analysis*. MetagenomeSeq analysis was used to compare the differences of all ASVs in the two groups of samples based on grouping, with |logFC|>1 and *P*<0.05 as the thresholds for screening significant differences (FC is fold change) to identify the significantly different microbial species.

In this experiment, different species were present among each experimental group and the control group, as shown in supplemental material. A total of 10 ASVs were significantly different in the CeO_2_-NPs groups with different ingestion doses compared with the control group, of which 7 belong to f_S24-7, 2 belong to g_Lactobacillus and 1 belongs to o_Clostridiales. Except for one ASV under f_S24-7, all showed significant abundance decreases after ingestion of CeO_2_-NPs. It is suggested that the alterations of f_S24-7, g_Lactobacillus and o_Clostridiales may be related to CeO_2_-NPs intake. In addition, the ASVs that were significantly changed in both the medium- and high- dose groups compared with the control group were significantly changed in addition to the 10 species mentioned above, with only one new ASV belonging to g_Streptococcus. Since Section 3.2.2 states that chronic enteritis occurred in the medium- and high-dose groups after ingestion of CeO_2_-NPs, we speculate that the presence of these ASVs may be associated with the development of enteritis.

## 4 Discussion

In this study, SD male rats were used as model animals to provide orally administered of CeO_2_-NPs of different doses (e.g., 0, 20, 100 and 500 mg/kg) for 90 days, during which no adverse reactions or deaths occurred with these rats. Meanwhile, the safety of CeO_2_-NPs was evaluated by pathology and second-generation sequencing, which is conducive to further elaborating the effects of CeO_2_-NPs on the intestinal tract and intestinal flora of rats. After exogenous substances are ingested orally, intestinal tissue is the site of their greatest exposure. Studies have shown that nanomaterials can aggravate or induce damage to intestinal tissue after oral exposure [24,25]. The study also reports that CeO_2_-NPs enter the blood circulation after ingestion, deposit in tissues and cause damage [26]. The pathology of this study shows that the ileum and cecum tissues of the rats in the medium- and high-dose groups suffered inflammatory damage, and the weight results show that the growth and development rates of rats in the medium- and high-dose groups slowed down in varying degrees. We believe that this may be because after CeO_2_-NPs enter the body through gastric filling, the nanoparticles cause a certain degree of damage to the intestinal tissue, affect the digestion and absorption of nutrients, destroy the barrier effect of intestinal epithelial cells on harmful substances, and then decrease the growth rate of rats.

Most of the nanoparticles are excreted through the digestive tract via the feces and come into direct contact with gut microorganisms to influence the flora structure of the intestinal microbe [27]. In this study, the overall structures of the intestinal flora of the experimental group and control group were not significantly different (*P*>0.05) from the perspective of the α-diversity and β-diversity. However, from the perspective of species compositions, the structures of the intestinal flora of rats in the control group and experimental group changed. Compared with the control group, the Mucispirillum and Akkermansia abundances increased after CeO_2_-NP ingestion. And, Mucispirillum was present only in the experimental group, and its abundance increased with increasing ingestion dose. The study suggests that the higher abundance of Mucispirillum is associated with the development of colitis and autoimmune diseases, and that is positively correlated with the incidence of enterocolitis [28,29]. Therefore, we hypothesize that the up-regulation of Mucispirillum might be associated with the development of enteritis in rats. Meanwhile, Akkermansia, a common intestinal probiotic, showed increased abundances after CeO_2_-NP intervention but decreased abundances with increasing doses of CeO_2_-NPs. The study indicates that Akkermansia can obtain energy material through secretion of mucus proteins [30,31], resist invasion of harmful bacteria through competitive inhibition, and enhance the integrity of intestinal epithelial cells by adhering to the surfaces of intestinal epithelial cells [32]. We therefore hypothesize that the increased abundance of Akkermansia found in this study may have resulted from a self-regulatory protective effect of the organism. Compared with the control group, the abundance of Weissella was significantly altered in the experimental group, as evidenced by a significant decrease in its abundance after ingestion of CeO_2_-NPs (*q*<0.05). Weissella is a constituent of normal intestinal flora, and only Weissella ceti is recognized as a harmful bacterium. The rest of the genus mostly have the characteristics of intestinal probiotics, such as fermentation of lactic acid, antagonism of harmful bacteria, and participation in the breakdown and utilization of fibrous food in the body [33,34]. Moreover, Weissella confusion was found to have a significant effect on the improvement of intestinal mucosal barrier function [35]. We therefore speculate that CeO_2_-NP ingestion reduced the abundance of Weissella, affected its protective effect on the intestinal mucosal barrier and allowed intestinal homeostasis to be disrupted, which in turn promoted the development of enteritis.

In addition, we also found that different doses of CeO_2_-NPs have different effects on intestinal flora, and the intestinal microecological balance may disrupt with increasing intake doses. In the low-dose group, the abundance significantly increased with the intestinal probiotics, Butyricimonas and Akkermansia, and significantly decreased with the pathogenic bacteria, Turicibacter, SMB53, Erythrobacter, and Flexispira. Akkermansia can resist the invasion of harmful bacteria through competitive inhibition, while Butyricimonas produce butyrate with anti-inflammatory properties to protect the intestines. Therefore, we speculate that intestinal inflammation was not significant in the low-dose group and may be related to changes of the intestinal probiotics, which maintained their own homeostasis. When CeO_2_-NPs were ingested in higher doses, the intestinal flora was significantly dysregulated, and the abundances of the intestinal pathogenic bacteria, Megasphaera, Mucispirllum, Desulfovibrio and Acinetobacter, were significantly increased in the medium- and high-dose groups and were mostly present only in the experimental group. Although the intestine correspondingly increased the expressions of the probiotics, Bifidobacterium and Anaerostipes, in the mid-/high-dose groups, we think that this increase may be more attributable to the repair of intestinal barrier action and inhibition of inflammation development after the onset of enteritis.

MetagenomeSeq analysis show that, compared with the control group, the abundances of f_S24-7, g_Lactobacillus and o_Clostridiales were significantly decreased, and the Streptococcus increased. S24-7, the main species of Bacteroides, is widely present in the intestinal flora of mice and has been shown to be the core flora that controls inflammation in the intestine by degrading complex carbohydrates, producing short-chain fatty acids, and scavenging free radicals [36,37]. Studies have shown that Lactobacillus, an intestinal probiotic, are the dominant bacteria in the intestine and can promote production of the anti-inflammatory cytokine, IL-10, to exert a protective effect [38]. Therefore, we suspect that CeO_2_-NPs mainly affect its role in the normal immune balance of the body by inhibiting expressions of species f_S24-7 and g_ Lactobacillus, which leads to inflammation in the body. Since most of the altered species are belong to f_S24-7, we infer that may be a targeted species after CeO_2_-NP ingestion. Streptococcus belongs to Pachylobacteria, which is a common pathogen in the body, and studies have suggested that it may be a transitional bacterium in the course from colitis to rectal cancer and exerts an injurious effect by activating inflammatory factors (e.g., NF-κB, TNF-α, IL-1, and COX-2) and the immune molecule, TLR2 [39,40]. we think that this increase after oral administration of CeO_2_-NPs may be related to enteritis.

The intestinal microbiota is a complex microbial community that colonizes the gastrointestinal tract, which can participate in a variety of physiological processes of the human body [41]. When the microecological balance inside the body will be broken, which can lead to imbalances in intestinal microflora, which can cause changes in the composition of intestinal flora or changes in metabolic pathways, thereby promoting the occurrence of a variety of diseases [42]. Therefore, based on the results of the pathology and second-generation sequencing in this experiment, we infer that the enteritis in rats may be associated with an alteration in the relative proportions of the pathogenic bacteria and intestinal probiotics. Decline in intestinal probiotics may be can reduced ability to defend against invasion by harmful bacteria, yet the mechanism of enteritis caused by the colonization of harmful bacteria needs to be further investigated.

## 5 Conclusion

In this experiment, the long-term effect of CeO_2_-NPs on the overall toxicity of the intestine was observed by using intragastric administration on rats and high-throughput sequencing technology with feces samples. It is found that long-term exposure to higher CeO_2_-NP doses (>100 mg/kg) could cause enteritis and changes in intestinal flora compositions. However, the results of existing studies do not yet make it clear as to whether the change in microflora is the cause or result of enteritis, which needs to be further confirmed. Whether the decreases in g_Mucispirillum, f_S24-7, g_Lactobacillus, and o_Clostridiales, and the increase in g_Weissella were associated with constant intake of CeO_2_-NPs, and whether the changes in their abundances can be used as indicators to monitor damage after CeO_2_-NP ingestion needs to be further explored. Therefore, it is necessary to further study the mechanisms of CeO_2_-NP intestinal microbiota interactions and build a CeO_2_-NP biosafety prediction and evaluation system.

## Supporting information

S1 Fig (PNG)

S2 Fig (PNG)

S3 Fig (PNG)

S1 File (TAR)
