# Correction: Effects of nano-cerium dioxide on intestinal microflora in rats by oral subchronic exposure

**DOI:** 10.1371/journal.pone.0304806

**Published:** 2024-05-30

**Authors:** Qianru Ye, Dantong Jia, Jun Ji, Yang Liu, Gang Wu

There are errors in the Funding statement. The correct Funding statement is as follows: This research was supported by the National Natural Science Foundation of China (Study on the effect of Nano Cerium Dioxide on Intestinal Microorganisms in Rats by long-term Exposure, No. 81860584).
