# Supplementary figures and images for: Effects of nano-cerium dioxide on intestinal microflora in rats by oral subchronic exposure
Source: PLoS One. 2024 Feb 29;19(2):e0298917. doi: 10.1371/journal.pone.0298917 (PMC10903844; doi:10.1371/journal.pone.0298917)

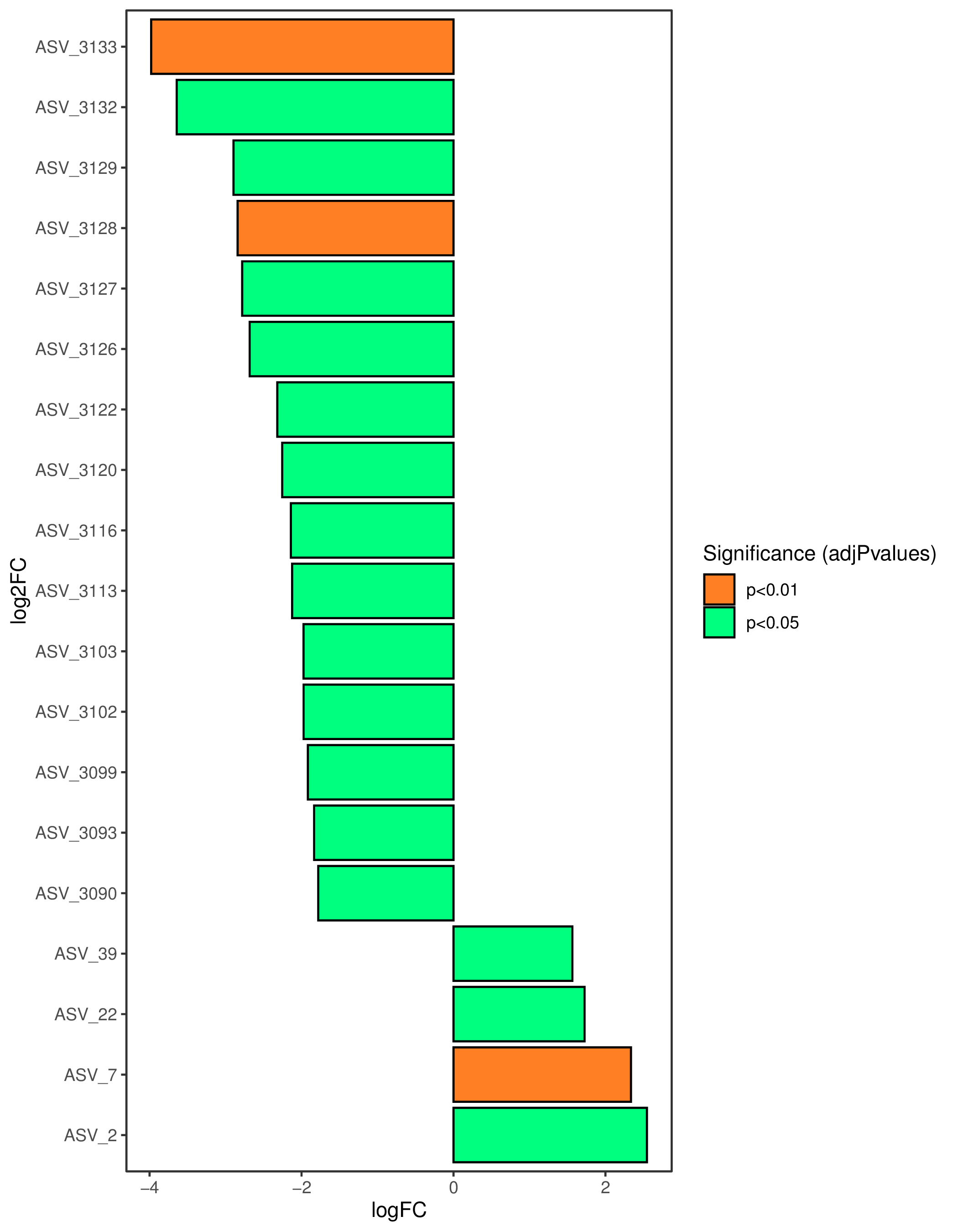

Supplement: S1 Fig — (PNG) [file pone.0298917.s001.png]

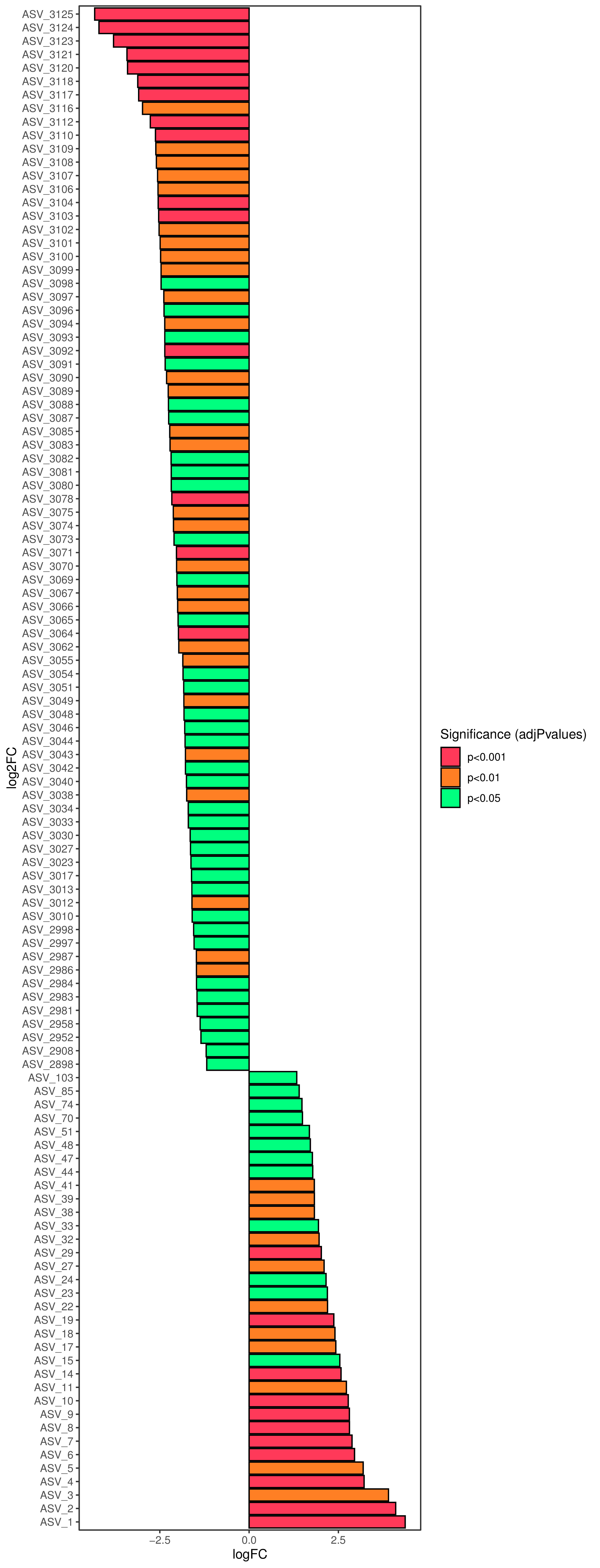

Supplement: S2 Fig — (PNG) [file pone.0298917.s002.png]

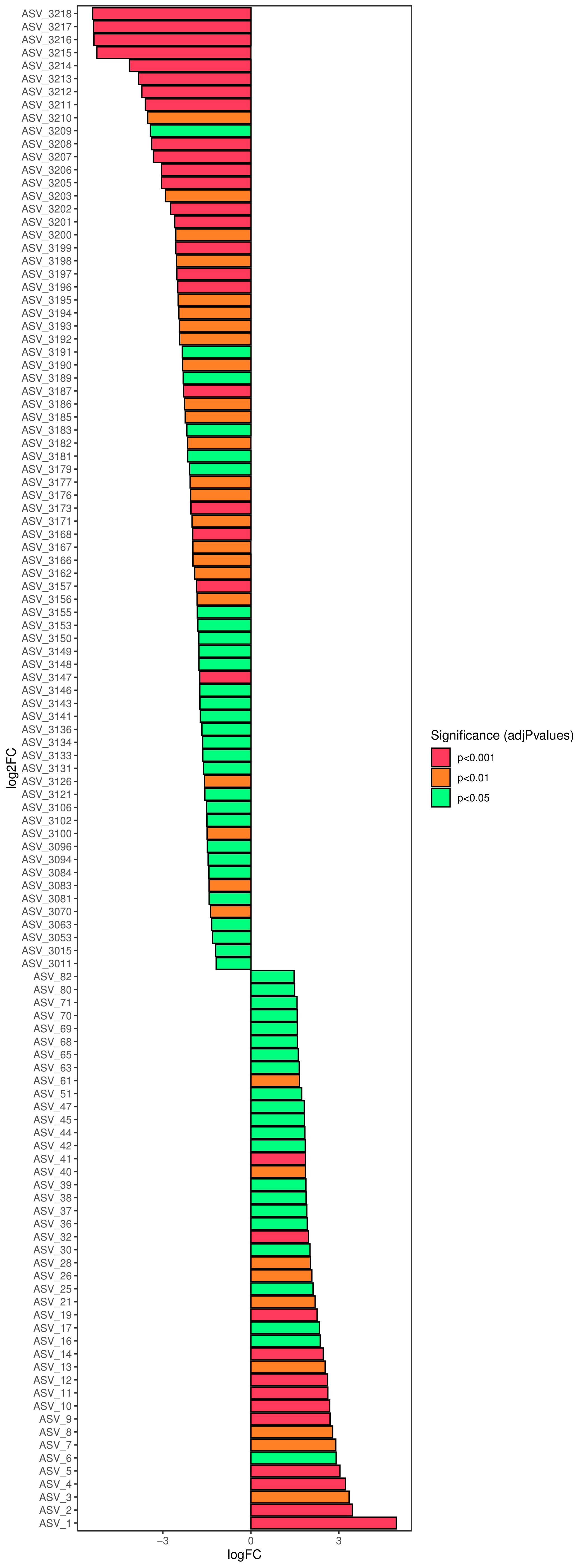

Supplement: S3 Fig — (PNG) [file pone.0298917.s003.png]
